# Paraneoplastic Cushing Syndrome Unmasking Small Cell Lung Cancer: A Rare Presentation

**DOI:** 10.7759/cureus.88382

**Published:** 2025-07-20

**Authors:** Jawad Malik, Laraib Arshad

**Affiliations:** 1 Respiratory Medicine, University Hospitals of Leicester NHS Trust, Leicester, GBR

**Keywords:** cushing's syndrome, ectopic cushing’s syndrome, metabolic alkalosis, non-diabetic hyperglycaemia, severe hypokalaemia, small-cell lung carcinoma

## Abstract

We present a case of a middle-aged woman who presented with chest pain and shortness of breath. Laboratory tests revealed persistent hypokalaemia, hyperglycaemia, and metabolic alkalosis despite treatment. Imaging identified a mass near the right hilum suggestive of lung malignancy. Endocrine evaluation showed markedly elevated cortisol and adrenocorticotropic hormone levels, consistent with paraneoplastic Cushing syndrome caused by ectopic hormone production. The analysis of the lung biopsy obtained through bronchoscopy confirmed the diagnosis of small cell lung cancer (SCLC). The patient was treated with metyrapone and spironolactone to stabilise her metabolic abnormalities and was subsequently referred for chemotherapy following a multidisciplinary team review. This case highlights the importance of recognising paraneoplastic syndromes as atypical presentations of malignancy and emphasises the role of a coordinated, multidisciplinary approach in diagnosis and management.

## Introduction

Paraneoplastic syndromes, although relatively uncommon, can serve as important early clues to an underlying cancer. One such rare and often overlooked condition is ectopic adrenocorticotropic hormone (ACTH) secretion, a form of paraneoplastic Cushing’s syndrome. This occurs when a non-pituitary tumor, most commonly small cell lung carcinoma (SCLC) or another neuroendocrine tumor, produces ACTH, leading to overstimulation of the adrenal glands and excessive cortisol production.

Unlike the more familiar presentation of Cushing’s syndrome, ectopic ACTH production tends to manifest with severe metabolic disturbances, such as persistent hypokalemia, metabolic alkalosis, hyperglycemia, and muscle weakness, often without the typical physical features like moon facies or central obesity. These atypical and rapidly progressing symptoms can delay diagnosis, especially in patients with aggressive malignancies.

A thorough diagnostic workup, including hormone assays, suppression testing, and imaging, is essential to pinpoint the source of ectopic hormone production. Early identification is critical, as the metabolic derangements associated with this syndrome can lead to significant morbidity if left untreated.

In this report, we present the case of a middle-aged woman whose initial symptoms of chest pain and shortness of breath led to the discovery of SCLC with ectopic ACTH production. Her case highlights the importance of considering paraneoplastic syndromes in the differential diagnosis of unexplained electrolyte abnormalities and metabolic dysfunction.

## Case presentation

We report the case of a 52-year-old Caucasian woman who presented with a one-week history of diffuse chest pain, progressive shortness of breath, and MRC dyspnea grade 1 initially and then progressing to grade 2. She had no prior history of similar symptoms, and her past medical history was unremarkable. On examination, there were no significant findings on systemic review.

Initial laboratory investigations revealed marked hypokalaemia, with a serum potassium level of 2.4 mmol/L, alongside significant hyperglycaemia (blood glucose: 20 mmol/L) and metabolic alkalosis (arterial pH: 7.52, bicarbonate: 32 mmol/L). Notably, the patient had no known history of diabetes mellitus (Table 1).

**Table 1:** Initial laboratory investigations This table summarizes the patient’s initial biochemical abnormalities, which include marked hypokalaemia, significant hyperglycaemia in the absence of known diabetes mellitus, and evidence of metabolic alkalosis on arterial blood gas analysis.

Parameter	Result	Reference range	Remarks
Serum potassium	2.4 mmol/L	3.5–5.0 mmol/L	Marked hypokalaemia
Blood glucose	20 mmol/L	3.9–7.8 mmol/L (fasting)	Significant hyperglycaemia; no known diabetes
Arterial pH	7.52	7.35–7.45	Metabolic alkalosis
Serum bicarbonate (HCO₃⁻)	32 mmol/L	22–28 mmol/L	Elevated, consistent with metabolic alkalosis

Despite intravenous and oral potassium supplementation, hypokalaemia persisted (Table 2). Hyperglycaemia also remained uncontrolled initially and was subsequently managed with insulin therapy.

**Table 2:** Daily serum potassium levels (day 1–day 7) This table presents serum potassium levels measured over a seven-day period, demonstrating persistently low values consistent with hypokalaemia despite Intra-venous and oral pottasium replacement.

Day	Serum potassium (mmol/L)	Reference range (mmol/L)
Day 1	2.4	3.5–5.0
Day 2	2.2	3.5–5.0
Day 3	2.8	3.5–5.0
Day 4	3.0	3.5–5.0
Day 5	2.9	3.5–5.0
Day 6	2.6	3.5–5.0
Day 7	2.7	3.5–5.0

**Figure 1:**
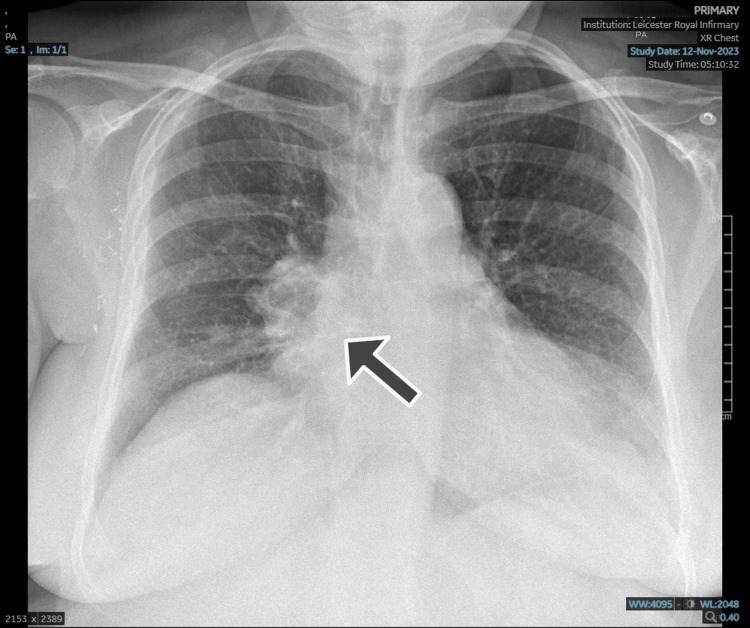
Initial chest X-ray Posteroanterior chest radiograph showing a spiculated opacity in the right mid-zone (black arrow), suggestive of a pulmonary mass. The lesion projects over the right hilum and may represent a primary bronchogenic carcinoma. No gross pleural effusion or pneumothorax is identified.

The patient presented with chest pain with respiratory symptoms, and an initial chest radiograph was suggestive of lung cancer (Figure 1). 

**Figure 2:**
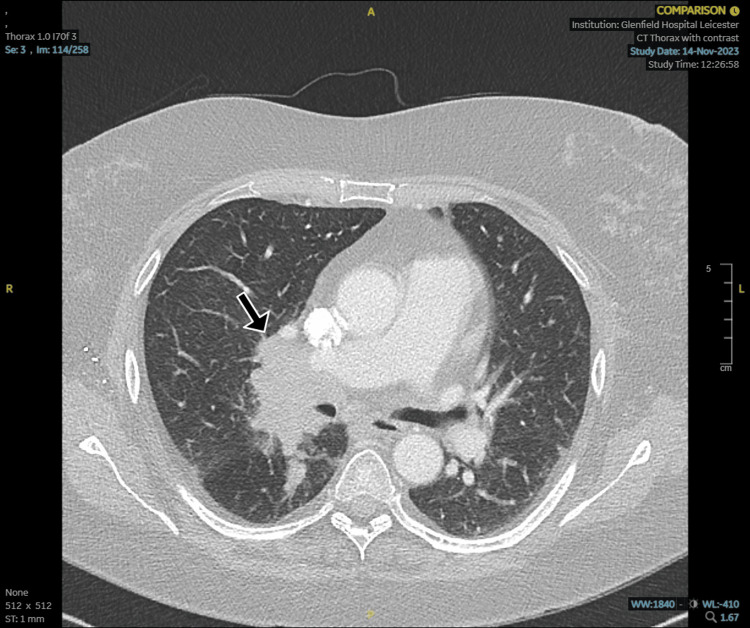
Computed tomography (CT) thorax Contrast-enhanced axial CT of the thorax demonstrating a spiculated right hilar mass (black arrow) measuring approximately 4 cm in greatest diameter. The mass is abutting the right main bronchus and associated with enlargement of adjacent mediastinal lymph nodes. No evidence of pleural effusion, chest wall invasion, or direct mediastinal involvement is seen on this image.

Further evaluation with contrast-enhanced CT of the thorax revealed a right hilar mass suspicious for a bronchogenic malignancy (Figure 2).

Given the persistent hypokalaemia, hyperglycaemia, and metabolic alkalosis, the possibility of a paraneoplastic endocrine syndrome was considered.

Endocrine workup showed markedly elevated serum cortisol levels (>2000 nmol/L), which failed to suppress following both low- and high-dose dexamethasone suppression tests. Plasma ACTH levels were also significantly elevated at 615 ng/L, consistent with ectopic ACTH secretion (Table 3).

**Table 3:** Endocrine workup results demonstrating elevated cortisol and adrenocorticotropic hormone (ACTH) levels with lack of suppression on dexamethasone testing Serum cortisol  levels during low- and high-dose dexamethasone suppression tests. Despite administration of both low- and high-dose dexamethasone, serum cortisol levels remained markedly elevated. Plasma ACTH was also significantly elevated at 615 ng/L, consistent with ectopic ACTH secretion. Reference ranges are included for comparison.

Test	Result	Reference range	Interpretation
Serum cortisol	>2000 nmol/L	Morning: 140–690 nmol/L	Markedly elevated
Low-dose dexamethasone suppression test	No suppression observed	Cortisol suppressed to <50 nmol/L	Abnormal; cortisol not suppressed
High-dose dexamethasone suppression test	No suppression observed	Cortisol suppressed by >50%	Abnormal; cortisol not suppressed
Plasma ACTH	615 ng/L	10–60 ng/L	Significantly elevated; ectopic ACTH secretion

**Figure 3:**
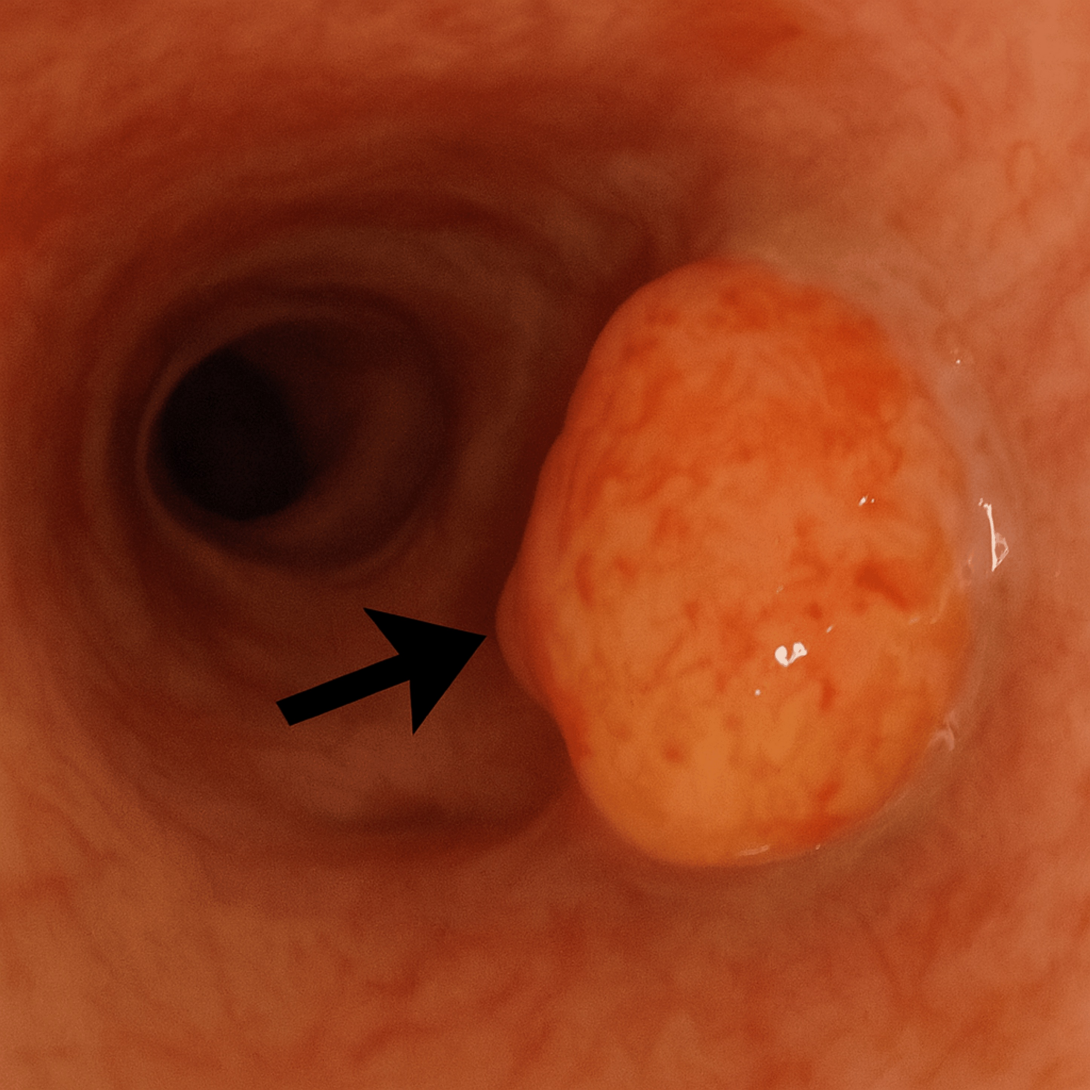
Bronchoscopic view of the right hilar mass Bronchoscopic view of the right bronchial tree demonstrating an irregular, lobulated endobronchial mass (black arrow). The lesion appears friable and hypervascular, partially obstructing the bronchial lumen, suggestive of a malignant endobronchial tumor.

**Figure 4:**
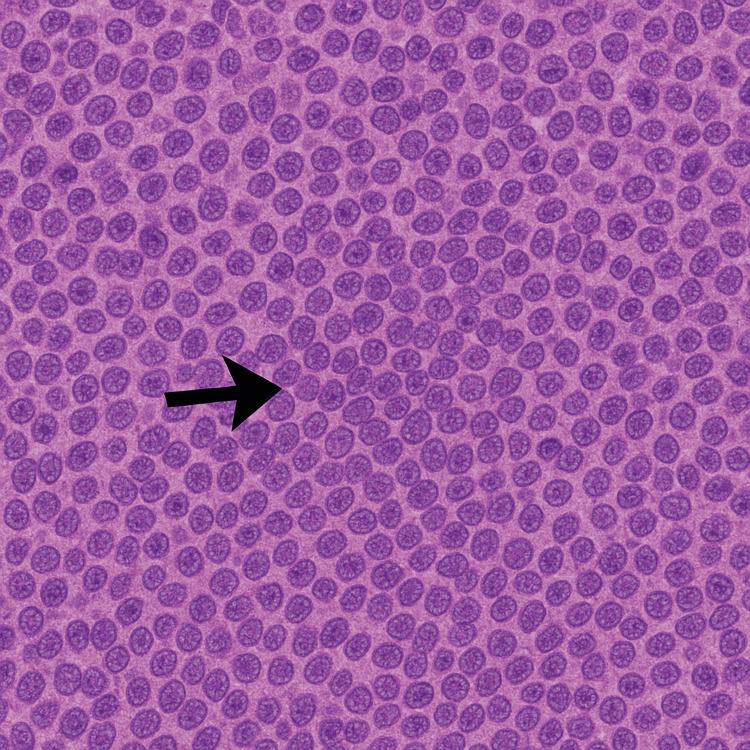
Histological section of small cell lung cancer (SCLC) The arrow indicates a dense cluster of small, hyperchromatic tumour cells characteristic for SCLC.

Flexible bronchoscopy was performed, and biopsy of the right endobronchial tumour confirmed the diagnosis of SCLC (Figures 3, 4).

The combination of persistent metabolic derangements, imaging findings, and histological confirmation supported the diagnosis of paraneoplastic Cushing’s syndrome secondary to ectopic ACTH production by SCLC. This rare clinical entity results from autonomous ACTH secretion by the tumour, leading to adrenal hyperplasia and excessive cortisol production.

Further staging workup was performed to assess the extent of the disease. Contrast-enhanced CT of the abdomen and MRI of the brain showed no evidence of distant metastasis. The disease was therefore classified as limited-stage SCLC.

**Figure 5:**
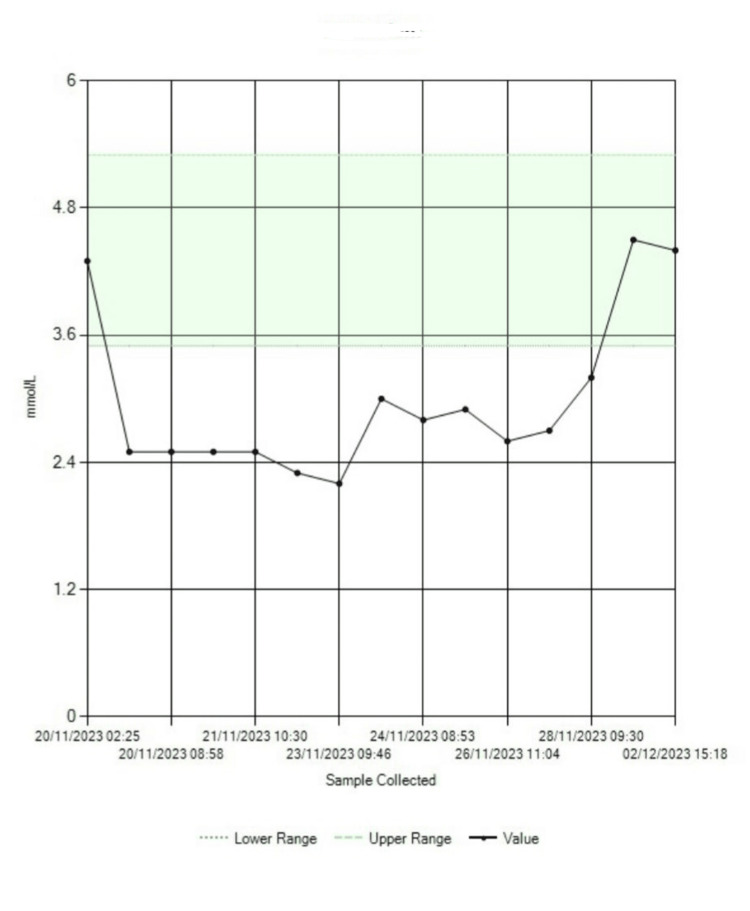
Serum potassium trend showing initial treatment resistance and subsequent stabilization after initiation of metyrapone and spironolactone The graph demonstrates persistently low serum potassium levels despite aggressive intravenous and oral supplementation. Notable stabilization and eventual normalization of potassium values are observed following the initiation of metyrapone and spironolactone, indicated toward the end of the monitoring period. The shaded green area represents the normal reference range for serum potassium (3.5–5.5 mmol/L).

The patient was commenced on metyrapone and spironolactone following a comprehensive discussion with the endocrinology team. This intervention resulted in the stabilisation of her potassium levels (Figure 5). Furthermore, in the context of her diagnosis of SCLC, a multidisciplinary team (MDT) was convened to discuss her case. Following this collaborative discourse, it was determined that a referral to the oncology department was warranted for the initiation of chemotherapy.

## Discussion

This case illustrates a rare but clinically significant presentation of paraneoplastic Cushing’s syndrome secondary to ectopic ACTH secretion from SCLC. The patient’s initial symptoms of chest pain and breathlessness were non-specific, but persistent metabolic derangements, including hypokalaemia, hyperglycaemia, and metabolic alkalosis, proved refractory to standard treatment. These findings raised suspicion for an underlying endocrine disorder, leading to targeted hormonal evaluation [1,2].

Diagnostic workup revealed markedly elevated cortisol and ACTH levels, with failure to suppress during low- and high-dose dexamethasone suppression tests. Imaging and histological analysis subsequently identified a right hilar mass consistent with SCLC as the source of ectopic ACTH production. Although rare, ectopic ACTH syndrome is a well-recognised paraneoplastic manifestation of SCLC, reported in approximately 1-5% of cases [3]. It can lead to severe metabolic derangements that complicate management and worsen prognosis if unrecognised [4].

Management of ectopic Cushing’s syndrome requires prompt biochemical stabilisation to mitigate life-threatening complications such as hypokalaemia and hypertension. In this case, metyrapone, an 11β-hydroxylase inhibitor, effectively reduced cortisol synthesis, while spironolactone antagonised mineralocorticoid receptors to correct hypokalaemia. Other agents such as ketoconazole, mitotane, or intravenous etomidate may be considered in similar cases, especially when rapid cortisol control is needed or oral therapy is contraindicated [1,5]. However, these therapies carry risks of hepatotoxicity, adrenal insufficiency, or sedation, underscoring the importance of careful monitoring.

Definitive treatment of the underlying malignancy remains the cornerstone of care, as sustained control of ectopic ACTH production depends on tumour response. Early initiation of chemotherapy in SCLC can lead to a reduction in tumour burden and, in some cases, resolution of the paraneoplastic syndrome [4]. However, the metabolic instability associated with hypercortisolism often complicates oncologic management, highlighting the need for coordinated multidisciplinary care.

This case underscores the diagnostic challenge posed by ectopic Cushing’s syndrome and the importance of recognising paraneoplastic endocrine presentations in patients with unexplained metabolic derangements. 

## Conclusions

This case underscores the importance of considering paraneoplastic syndromes in patients with persistent, unexplained metabolic derangements such as hypokalaemia, hyperglycaemia, and metabolic alkalosis. In this patient, early recognition of ectopic ACTH secretion prompted targeted investigations, leading to the timely diagnosis of SCLC. This facilitated the initiation of appropriate endocrine therapy with metyrapone and spironolactone to stabilise the biochemical abnormalities and allowed safe progression to oncological management.

The case also highlights the complexities of managing ectopic Cushing’s syndrome, where severe metabolic disturbances can delay definitive cancer treatment. A coordinated, multidisciplinary approach involving endocrinology, oncology, and respiratory teams was crucial in optimising patient care and improving the likelihood of a favourable outcome.

For clinicians, this case reinforces the need to maintain a high index of suspicion for paraneoplastic endocrine disorders in patients with unexplained electrolyte and metabolic abnormalities, particularly when accompanied by respiratory symptoms or imaging suggestive of a pulmonary lesion. Early identification and intervention in such cases are critical for minimising morbidity and enabling timely cancer-directed therapy.
